# Growth hormone releasing peptide-6 (GHRP-6) prevents doxorubicin-induced myocardial and extra-myocardial damages by activating prosurvival mechanisms

**DOI:** 10.3389/fphar.2024.1402138

**Published:** 2024-05-30

**Authors:** Jorge Berlanga-Acosta, Danay Cibrian, Juan Valiente-Mustelier, José Suárez-Alba, Ariana García-Ojalvo, Viviana Falcón-Cama, Baohong Jiang, Linlin Wang, Gerardo Guillén-Nieto

**Affiliations:** ^1^ Center for Genetic Engineering and Biotechnology, Playa, Cuba; ^2^ Institute of Cardiology and Cardiovascular Surgery, Havana, Cuba; ^3^ Shanghai Institute of Materia Medica, Chinese Academy of Sciences, Shanghai, China

**Keywords:** doxorubicin, dilated cardiomyopathy, GHRP-6, heart failure, ventricular dilation

## Abstract

**Introduction:** Dilated cardiomyopathy (DCM) is a fatal myocardial condition with ventricular structural changes and functional deficits, leading to systolic dysfunction and heart failure (HF). DCM is a frequent complication in oncologic patients receiving Doxorubicin (Dox). Dox is a highly cardiotoxic drug, whereas its damaging spectrum affects most of the organs by multiple pathogenic cascades. Experimentally reproduced DCM/HF through Dox administrations has shed light on the pathogenic drivers of cardiotoxicity. Growth hormone (GH) releasing peptide 6 (GHRP-6) is a GH secretagogue with expanding and promising cardioprotective pharmacological properties. Here we examined whether GHRP-6 administration concomitant to Dox prevented the onset of DCM/HF and multiple organs damages in otherwise healthy rats.

**Methods:** Myocardial changes were sequentially evaluated by transthoracic echocardiography. Autopsy was conducted at the end of the administration period when ventricular dilation was established. Semiquantitative histopathologic study included heart and other internal organs samples. Myocardial tissue fragments were also addressed for electron microscopy study, and characterization of the transcriptional expression ratio between Bcl-2 and Bax. Serum samples were destined for REDOX system balance assessment.

**Results and discussion:** GHRP-6 administration in parallel to Dox prevented myocardial fibers consumption and ventricular dilation, accounting for an effective preservation of the LV systolic function. GHRP-6 also attenuated extracardiac toxicity preserving epithelial organs integrity, inhibiting interstitial fibrosis, and ultimately reducing morbidity and mortality. Mechanistically, GHRP-6 proved to sustain cellular antioxidant defense, upregulate prosurvival gene Bcl-2, and preserve cardiomyocyte mitochondrial integrity. These evidences contribute to pave potential avenues for the clinical use of GHRP-6 in Dox-treated subjects.

## Introduction

Dilated cardiomyopathy (DCM) is a group of heterogeneous myocardial diseases, with structural and functional disorders defined by left ventricular (LV) or biventricular dilation, along with systolic dysfunction with abnormal left ventricle ejection fraction (LVEF) (Schultheiss et al., 2019). DCM pathophysiological changes include a decrease in stroke volume and cardiac output, impaired ventricular filling and an increase in end-diastolic pressure. Diastolic function is also impaired accounting for a reduction in myocardial relaxation, and consequently a poor ventricular filling (Riehle and Bauersachs, 2019; Schultheiss et al., 2019). This is a common and highly prevalent condition leading to heart failure (HF) (Hershberger et al., 2013). Despite the remarkable progress in HF control therapies over recent decades, DCM mortality rates are high, remaining as one of the leading causes of heart transplantation (Benjamin et al., 2018; Ferreira et al., 2023).

Genetic mutations are reported to account for 35% of DCM cases, whereas other lifetime acquired causes include viral myocarditis, toxins, endocrine-metabolic disturbances, and exposure to chemotherapy drugs in cancer-affected patients (Imanaka-Yoshida, 2020). Doxorubicin (Dox) is a chemotherapeutic anthracycline with proved efficacy against different cancer types but with remarkable cardiotoxicity. DCM and progressive HF are frequently-registered adversities in Dox-treated patients (Robert Li et al., 2023). Hence, different cardiovascular-damage drivers have been attributed to Dox, including myocardial cells DNA damage, interstitial inflammation, oxidative stress cytotoxicity, cardiomyocytes apoptosis, mitochondrial damages, and dysregulation of autophagy (Wang T-H. et al., 2023). Dox toxicity is not solely restricted to myocardial tissue; its cytotoxic effects impact a broad constellation of epithelial, mesenchymal, and nervous cells, affecting most organ systems (Alhowail et al., 2019; Prathumsap et al., 2020). Dox at different doses and treatment regimens has been for years a valuable tool for the experimental reproduction of DCM/HF, which has contributed to elucidate molecular pathogenic determinants of its underlying cardiotoxicity (Hullin et al., 2018; Lother et al., 2018; Zhu et al., 2019).

Growth hormone (GH) releasing peptide 6 (GHRP-6) (His-DTrp-Ala-Trp-DPhe-Lys-NH2) is a small molecular weight peptide integrated into the GHRP family, which has progressively broadened its pharmacological spectrum from a GH-secretagogue to a promising cardioprotective agent (Berlanga-Acosta et al., 2017). This agent is a ghrelin analog that binds and activates the GH secretagogue receptor 1a (GHSR1a) (Xiao et al., 2020), whereas it also binds to the ectodomain of CD36 receptor (Demers et al., 2004).

GHRP-6 has proved to prevent and attenuate cardiac cell death and LV failure in a variety of experimental scenarios (Lucchesi, 2004; Xu et al., 2005; Berlanga-Acosta et al., 2016; Berlanga-Acosta et al., 2017). Furthermore, we have also identified GHRP-6 anti-fibrotic properties which may contribute to mitigate the systemic complications of Dox administration (Berlanga-Acosta et al., 2012; Mendoza et al., 2016; Fernandez-Mayola et al., 2018). Beyond its ability to enhance the survival of a diversity of cells before an otherwise lethal stress, GHRP-6 and other mimetic ligands to the GHSR1a and CD36 receptors, play an agonistic effect on the GH/IGF-1 axis promoting a systemic anabolic response, and counterbalancing catabolism and sarcopenia (Giorgioni et al., 2022).

Here we describe that: (Schultheiss et al., 2019): GHRP-6 administration concomitant to Dox challenge prevented the onset of DCM/HF, (Riehle and Bauersachs, 2019), GHRP-6 significantly reduced animals’ morbidity and mortality, attenuated epithelial damages in a multi-organs spectrum, and inhibited Dox-related parenchymal fibrotic induration, (Hershberger et al., 2013), GHRP-6 triggered multiple defense mechanisms, involving oxidative stress reduction and activation of detoxifying enzymes, enhancement of prosurvival gene expression, and cardiomyocytes mitochondrial structural preservation. To the best of our knowledge, this is the first demonstration on the GHRP-6 cardio and systemic protective and anti-fibrotic effects, in the scenario of Dox-related toxicity.

## Materials and methods

### Animals and ethics

Male Wistar rats with 200–250 g body weight and 9–10 weeks of age were used for the study. Rats were purchased from the National Center for Laboratory Animals Breeding (CENPALAB) and housed in a certified room of the animal facility at the Center for Genetic Engineering and Biotechnology (CIGB). Three animals per cage were allocated under controlled environmental conditions. Rats were allowed free access to food and water. Animals’ manipulation, care, and investigational procedures were declared in the experimental protocols and approved by the Institutional Animal Care and Welfare Committee of CIGB. Orbital blood samples were obtained under ether anesthesia. Animals were terminated by anesthesia overdose (250 mg/kg sodium pentobarbital).

### Reagents, treatments, and DCM induction

**FIGURE 1:**
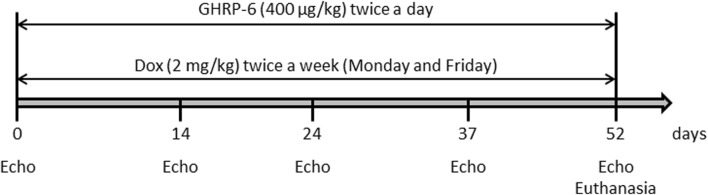
Schematic representation of the experimental induction of DCM/HF in rats by intraperitoneal administration of Dox, and concomitant administration of GHRP-6.

Medical grade, commercially injectable solution doxorubicin hydrochloride (Dox) was acquired from Lemery SA pharmaceutical company (Mexico) at a concentration of 2 mg/mL. DCM/HF pathological model was induced through the intraperitoneal administration of Dox at 2 mg/kg twice a week (Monday and Friday), for 52 days as described (Hayward and Hydock, 2007) (Figure 1). The hexapeptide GHRP-6 (His-d-Trp-Ala-Trp-d-Phe-Lys-NH2) was purchased from BCN Peptides (Barcelona, Spain). Fresh preparations were obtained by diluting the peptide in sterile normal saline solution to a final concentration of 400 μg/mL. Solutions were freshly prepared, conserved at 4°C and protected from light. GHRP-6 was intraperitoneally administered at a dose of 400 μg/kg.

### Experimental protocols and study groups

The hypothesis for this study was that GHRP-6 concomitant administration to Dox treatment may attenuate myocardial structural and functional damages, and consequently, prevent the onset of DCM/HF. A total of 36 animals were used, distributed in three experimental groups with 12 rats each: (I)—A group of healthy not treated, sentinel animals was used to obtain echocardiographic and biochemical reference values at the end of the intoxication period. This is relevant considering that rats’ somatic maturation was still in progress during the study period (Cossio-Bolanos et al., 2013). (II)- Animals receiving GHRP-6 twice a day at a dose of 400 μg/kg concomitant to Dox. (III)- Animals receiving normal saline solution concomitant to Dox. Following to experimental groups formation and prior to Dox-administration regimen, all the rats were echocardiographically studied in order to obtain baseline physiologic parameters. Subsequent serial echocardiographic evaluations were done during the Dox administration process on the following points: 14, 24, 37, and 52 days (Figure 1). For the later point, the rats had a Dox cumulative dose of 30 mg/kg and echocardiographic recordings indicated a clear LV deformity and functional failure, as described in previous studies (To et al., 2003). As mentioned, treatments with Dox and GHRP-6 were intraperitoneally administered until day 52 (see Figure 1 for experimental sequence).

### Echocardiographic parameters

Prior to each echocardiography recording, animals were lightly sedated as recommended (Stein et al., 2007) with an intraperitoneal administration of ketamine (50 mg/kg) + xylazine (5 mg/kg), and placed in supine position in a stable horizontal plane. Once the animals were sedated and stabilized, transthoracic echocardiography recordings were carried out with a Sonos 5,500 equipment, coupled to a linear transducer of 11–15 MHz (Philips, United States). The M-mode structural parameters studied were: LV diastolic diameter (LVDd), LV systolic diameter (LVSd), inter-ventricular septum thickness in systole (IVSs), and LV posterior wall thickness in systole (LVWs). LVEF was considered the main functional parameter as previously indicated (Iwase et al., 2004).

### Histopathologic evaluations

Animals were euthanized by anesthesia overdose at the end of the administration period. Alternatively, the rats that along the study evolved to a terminal irreversible clinical condition were sacrificed to ensure a proper autopsy and samples collection. Accordingly, these cases were included in the mortality record. Autopsy study was conducted following an internal protocol based on standard techniques (Scudamore et al., 2014). During the autopsies, gross pathological changes were described and recorded. Heart, lungs, and liver weights were used to calculate the relative organs weight indexes as described: Relative organ weight = [organ weight/body weight]×100 (Mossa et al., 2015). Representative fragments from apparently normal organs were also harvested. Hearts were sliced in four sagittal sections from apex to base as described (Li et al., 2019). Organs fragments and heart slices were 10% buffered formalin fixed and processed for paraffin embedding. Semi-thin sections (2–3 µm) were serially generated for subsequent hematoxylin/eosin staining. Semi-quantitative histological analyses of the heart, bronchial mucosa, kidneys, and liver passive congestion (Table 1) were conducted according to previous descriptions (Chen et al., 2016; Afsar et al., 2017). Histological images were obtained using an Olympus BX-53 light microscope (Olympus America Inc., United States) whereas the histological evaluations were independently and blindly performed by specialized researchers (JBA, DCV, JSA).

**TABLE 1:** Histological examination and damage scoring parameters.

Organ evaluated	Parameter evaluated	Evaluation procedure
Myocardial LV wall	% of damaged myofibrils. Damage criteria were: presence of granular basophilic material, loss of fibers, fiber thinning, tumefaction, fragmentation and reduction of eosin staining affinity	Ten random microscopic fields of longitudinal fibers (×20) were studied/animal. The % of damaged fibers was determined, considering the total number of fibers evaluated in each field
Lungs	% of bronchi with mucosal necrosis	Ten random microscopic fields (×10) were studied per animal. The % of damaged bronchi was determined considering the total in each field
Kidneys	% of tubules with irreversible epithelial cells damages	Ten microscopic fields (×20) were studied per animal. The % of tubules irreversibly damaged was determined, out of the total in each field
Liver	Passive hepatic congestion	0- No congestion, 1- Mild congestion, 2- Evident congestion, 3- Severe congestion with a marked sinusoidal distortion

### Electron microscopy study

Specimens were fixed in 3.2% glutaraldehyde and post-fixed in 1% osmium tetroxide, at 4°C and for 60 min, washed with PBS (0.1 M; pH 7.2) and dehydrated in increasing concentrations of ethanol at 4°C. Inclusion was carried out as described (Glauert and Lewis, 1999). Ultrathin sections obtained with an ultramicrotome (NOVA, LKB, Germany), 400–500 Å thick; were placed on 400-hole copper-nickel grids, counterstained with saturated uranyl acetate and lead citrate, and subsequently examined with a JEOL JEM 2000 EX microscope (JEOL, Japan). A total of 20 photomicrographs at different magnifications of each sample collected were blindly analyzed by a qualified specialist (VFC). The presence of pathological changes in the ultrastructure of myofibrils, mitochondria, and intercellular junctions were qualitatively evaluated.

### Serum biochemical analysis

Blood was collected from the retro-orbital plexus once the animals were anesthetized. Blood was centrifuged at 10,000 rpm for 15 min at 4°C to obtained serum. Serum samples were aliquoted and kept at −80°C for subsequent analysis. The concentration of total hydroperoxides (THP), malondialdehyde (MDA), and the activity of the enzymes superoxide dismutase (SOD) and catalase were determined using an UV/visible spectrophotometer Ultrospec 2000 (Pharmacia Biotech, United States). The quantification of THP and MDA contents was made using commercial kits Bioxytech H2O2-560 and Bioxytech LPO-586 (OXIS International Inc., United States), respectively, following the manufacturer’s instructions. Total SOD activity was evaluated by the classic method based on pyrogallol autoxidation as described (Rao et al., 2021). We consider 1 U of SOD as the amount of enzyme inhibiting 50% of pyrogallol autoxidation reaction at 25°C. Catalase activity was determined following the decomposition of the H_2_0_2_ at 240 nm in 10-s intervals for 1 min (Ajamieh et al., 2002). Alanine amino transferase (ALAT) serum levels were measured in an automatic analyzer (Hitachi 747, Germany), according to the manufacturer’s instructions.

### Gene expression study

In order to evaluate the expression ratio between Bcl-2 and Bax as representative of pro-survival and apoptogenic gene, respectively; LV tissue fragments were collected from five rats of saline and GHRP-6 groups during the autopsy process. Fragments were stored in liquid nitrogen until processing. Total RNA purification was performed by treatment with Tri-Reagent (Sigma-Aldrich, United States), following the manufacturers’ instructions. The quality and quantity of the purified RNA was estimated by determining the absorbance at 260 nm and 280 nm, together with the visualization by electrophoresis of the two ribosomal RNA bands. The contaminating genomic DNA was removed by digestion with the enzyme DNaseI (Epicentre Technologie, United States), free of RNase activity, following the manufacturers’ instructions. An amount of 1 μg of the RNA previously treated with DNaseI was used for the RT-PCR technique, using the commercial GeneAmp RNA PCR Core Kit (Applied Biosystems, United States), according to the manufacturers’ specifications. The amplification of the genes of interest was carried out using the primers and temperatures specified below (Table 2). β-actin gene expression was used to normalize the expression of the target genes. The bands obtained were detected by applying 1/10 or 1/5 of the volume of the amplification reaction in a 1% agarose gel electrophoresis. The intensity of the bands was quantified using Kodak ID 3.6 software (Kodak, United States).

**TABLE 2:** Primer sequences and amplification conditions.

Gen	Primers sequence	GenBank number	PCR Temp. (°C)	PCR product length (bp)
Bax	5′- TGA TTG CTG ACG TGG ACA CGG AC	236U23	68/94	321
	3′- TGA GCG AGG CGG TGA GGA CTC			
β-actin	5′-GGA GAT CGT GCG GGA CAT CAA GG	AY550069	68/94	482
	3′-GGC CGG ACT CGT CGT ACT CCT GC			
Bcl-2	5′- GCT ACC GTC GCG ACT TTG CAG AG	545U23	68/94	321
	3′- CAC TTG TGG CCC AGG TAT GCA CC			

### Statistical analyses

The statistical analyses were made with GraphPad Prism (California, United States), version 8.0.2. The Goodness of Fit tests to the normal distribution (Kolmogorov-Smirnov test) were made for all data, as well as the homogeneity of variances (Bartlett test). Given that in all cases the assumptions of normal distribution and homogeneity of variances were fulfilled, we carried out comparisons between pairs of groups using the Student’s *t*-test. For multiple comparisons, we used one-way or two-way analyses of variance (ANOVA) followed by the Newman Keuls, Sidak’s or Dunnett’s multiple comparisons tests. The results of survival were compared through the Logrank test. In all cases, the values of *p* < 0.05 were interpreted as indicative of statistically significant differences.

## Results

### Clinical evolution and mortality

Having completed 3 weeks of Dox administration, animals began to show a cachectic process evolving to clinical deterioration. Rats from both GHRP-6 and saline groups showed no differences in body weight loss along the Dox administration period (Table 3, *p* = 0.1943), which significantly differed to the body weights recorded for the healthy sentinel rats (*p* < 0.0001) on day 52.

**TABLE 3:** Body weight of the animals and relative organs weight index.

Groups	Body weight (g)	W.I. Heart	W.I. Lungs	W.I. Liver
Healthy sentinel	417.2 ± 27.2 (a)	0.28 ± 0.03 (b)	0.41 ± 0.07 (b)	3.54 ± 0.33 (c)
Saline	266.6 ± 32.5 (b)	0.51 ± 0.08 (a)	1.02 ± 0.28 (a)	6.79 ± 0.33 (a)
GHRP-6	295.5 ± 45.1 (b)	0.33 ± 0.05 (b)	0.62 ± 0.16 (b)	4.96 ± 0.51 (b)

The values are represented as the mean ± SD, per experimental group. The statistical analysis was made using an ANOVA, followed by the Newman-Keuls multiple comparison test. For each parameter, different letters (a, b or c) indicate statistically significant differences among the experimental groups, while equal letters indicate not significant differences, *p* < 0.05. W.I.: relative weight index.

**FIGURE 2:**
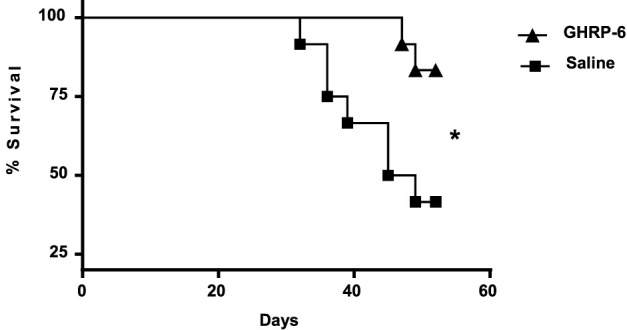
Survival curve in doxorubicin-treated animals. GHRP-6 and saline experimental groups started the study with *n* = 12 rats per group. The statistical analysis was carried out using the Logrank test. *p = 0.0230.

Tendency to isolation, prostration, bristly hair, and dorsal hunched posture was observed in most of the animals exposed to Dox. Autopsies showed that GHRP-6 intervention significantly reduced the relative weights of the heart and lungs as compared to saline (all *p* < 0.05). No statistical difference was detected in the relative weights of the heart and lungs from the GHRP-6 intervention, and those of the healthy sentinel animals (Table 3). Most importantly, GHRP-6 intervention allowed for a significant survival percentage (84%) as compared to saline group (42%), (Figure 2).

### GHRP-6 contributed to preserve ventricular morphology and physiology

**FIGURE 3:**
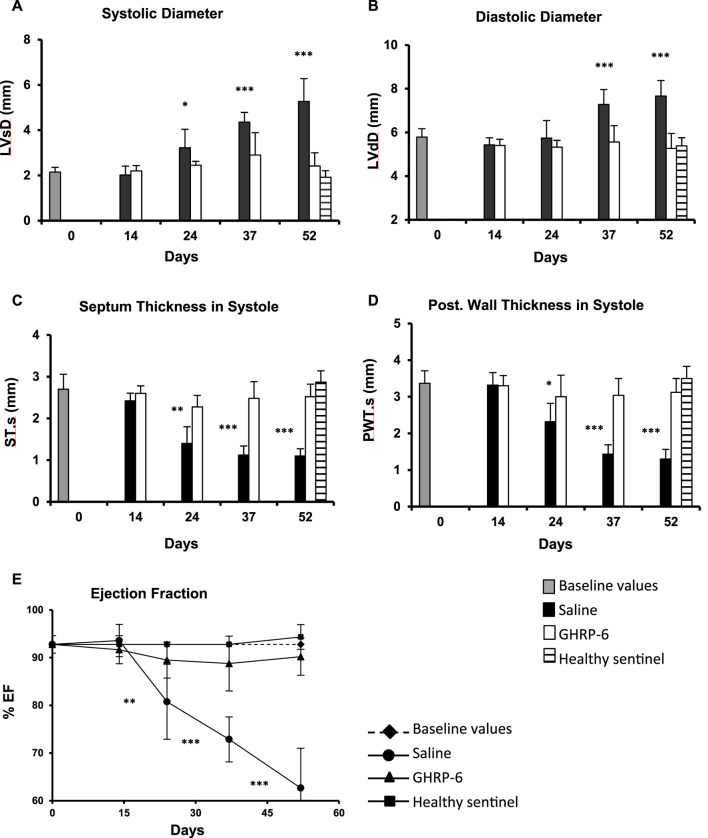
Echocardiographic characterization of morphologic and functional parameters in healthy and doxorubicin-treated animals. **(A)** Systolic diameter. **(B)** Diastolic diameter. **(C)** Septum thickness in systole. **(D)** Posterior wall thickness in systole. **(E)** LV ejection fraction. The data are represented as the mean ± SD per experimental group. The baseline values correspond to pre-Dox administration phase. The statistical analyses were made using the unpaired Student’s *t*-test. *p < 0.05; **p < 0.01; ***p < 0.001.

The rats included in the study exhibited normal morphologic and functional baseline echocardiographic parameters. Average baseline LVEF recording was 92.78% ± 1.84%. The earliest myocardial morphological change in the saline group was detected on day 24th of the experiment, given by a significant increase (*p* = 0.0279) in the LV systolic diameter, (Figure 3A), and followed by a significantly larger LV diastolic diameter after 37 days of Dox administration (*p* < 0.0001) (Figure 3B). These impairments appeared concomitant to a significant thinning of the septum and the posterior wall in systole (*p* < 0.05) (Figures 3C, D) in relation to the baseline data. The myocardial structural deterioration translated in a significant reduction of the LVEF in the saline group when compared to its baseline values. Significant differences were also observed when the saline group LVEF was compared to the healthy sentinel and GHRP-6 groups. These alterations were progressively worse in correspondence with Dox cumulative dose. On day 52, saline group exhibited a decrease of about 30% in LVEF (Figure 3E). Concomitant GHRP-6 intervention prevented LV dilation regardless of the dose of Dox. Accordingly, no statistical differences in the diastolic and systolic diameters were detected in reference to its baseline data, and as compared to the healthy sentinel animals on day 52 (Figures 3A, B). Furthermore, GHRP-6 treated group also showed septal systolic thickness and posterior wall thickness values, similar to those measured in the healthy sentinel group (Figures 3C, D). Worth mentioning is that GHRP-6 group LVEF figures, remained within the normal range during the entire evaluation period as compared to the healthy sentinel group recordings (Figure 3E, all *p* > 0.05).

### GHRP-6 intervention protected ventricular myofibrils from Dox toxicity

**FIGURE 4:**
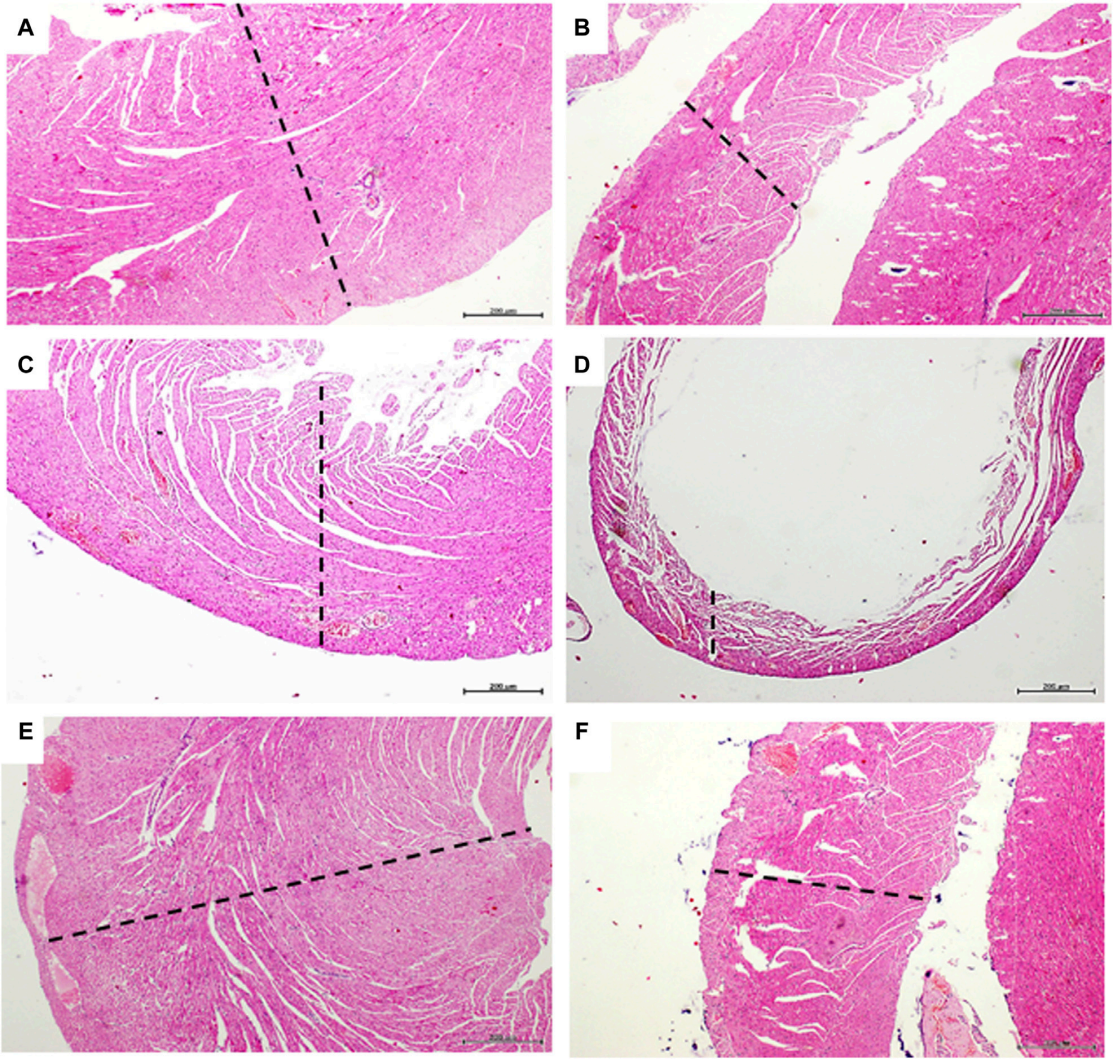
Myocardial histologic aspect of healthy and doxorubicin-treated rats. Microphotographs in the left column correspond to left ventricle. The right ventricle images are on the right column. **(A,B)** are representative images of left and right ventricle walls of animals from the healthy sentinel group where no evidence of pathologic changes were found. Tissue mass and fibers compactness are normal. **(C,D)** are images representing the major histopathologic damage brought about by Dox treatment in the saline group. It is evident the “loss-of-substance” by cardiomyocytes demise with evident wall thinning of both left and right ventricular walls. **(E,F)** are images representing the cardioprotective effect of GHRP-6. Myocardial cells demise and loss of ventricular tissue mass by Dox were evidently prevented by the concomitant intervention with the peptide. Dashed lines are introduced to facilitate the transmural view of ventricular walls. Autopsy collected samples and paraffin processing for semi-thin sections stained with H/E. For all, magnification is ×4. Scale bar is 200 µm.

Figures 4A, B correspond to panoramic images of both ventricles free walls of rats from the healthy sentinel group, in which no histopathological damages were detected. As expected, Dox treatment produced a group of myocardial abnormalities on the saline-treated rats that encompassed: myofibrils thinning, wall slimming (Figures 4C, D), fibers undulation, fractures, and focal loss of eosin staining affinity. GHRP-6 administration however, exerted a “sparing effect” on the ventricular myofibrils against Dox-induced damages. Myocardial histopathological damages attributable to Dox were minimal, whereas no microscopic differences were detected between GHRP-6 rats and the intact sentinel animals (Figures 4E, F). This microscopic cardioprotective effect was further supported by the quantitative morphometric analysis, attesting a significant reduction (*p* < 0.00019) in the percentage of damaged ventricular myofibrils (Saline: 91.0 ± 4.4 vs. GHRP-6: 42.2 ± 8.6).

### GHRP-6 also protected epithelial extra-cardiac organs

**FIGURE 5:**
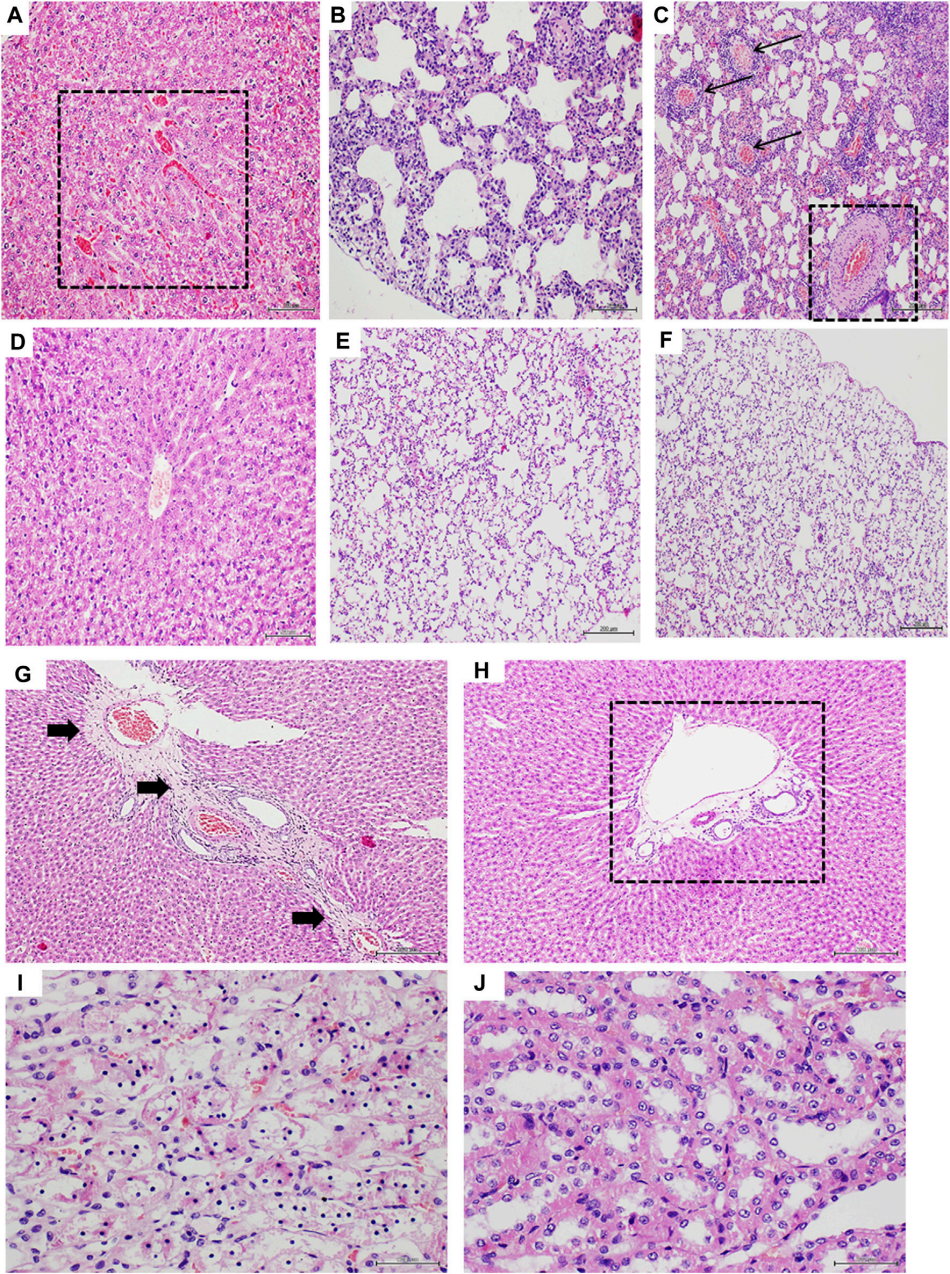
Histologic characterization of doxorubicin-induced extracardiac damages. **(A–C)** correspond to liver and lung parenchyma of saline group. **(D–F)** are representative of the effect of GHRP-6 treatment in these organs. **(A)** Liver section exhibiting passive congestion given by sinusoidal blood congestion (square). Magnification ×20. **(B)** is representative of Dox-associated lung damage, expressed by a dramatic alveolar walls thickening and mixed hypercellularity, including round inflammatory cells. Magnification ×20. **(C)** Shows the intense vessels congestion in lungs of control saline-treated rats. The arrows point to congested veins, and the square to a congested artery with a remarkable wall thickening. Note also the alveolar walls congestion and hypercellularity. Magnification ×10. **(D)** Image representative of a liver with no venular/sinusoidal congestion in animals treated with GHRP-6. Magnification ×20. **(E)** The image shows thin and normo-cellular alveolar walls with no collapse or reduction of the functional intra-alveolar space. Magnification ×20. **(F)** Image of a normal lung parenchyma with no vascular congestion nor infiltration of inflammatory cells in animals receiving GHRP-6. Magnification ×10. **(G)** is a representative image of the fibrotic process triggered by the Dox treatment in the liver of saline control group. Black arrows indicate the long trajectory of a thick fibrotic cord that crosses around veins. Magnification ×10. **(H)** The image shows the effect of GHRP-6 in preventing liver perivascular fibrosis. The square remarks the presence of normal, no fibrotic matrix around a central vein. Magnification ×10. **(I)** is a high magnification (×40) microphotograph of a kidney tubular system of control saline animals showing massive nuclear pyknosis and cytoplasmic ballooning, all irreversible lethal changes. **(J)** The image shows that GHRP-6 intervention exerted a nephro-protective effect, preventing degenerative and lethal nuclear and cytoplasmic damages. Magnification ×40. All are semi-thin sections, H/E staining. Scale bar is 200 µm.

The histological examination of the lungs and liver parenchyma of animals receiving Dox and saline solution revealed pathognomonic changes of congestive heart failure (CHF). Passive liver congestion; as lungs alveolar septal thickening, edema, hypercellularity, and venous congestion were largely prevalent in the saline group (Figures 5A–C). All these pathological findings were partially or absolutely prevented by the intervention with GHRP-6 (Figures 5D–F). These histopathologic changes were substantiated by semiquantitative measurement scales as described in Table 1. Liver congestion in saline group was classified as evident-to-severe versus less than mild congestion for the GHRP-6 group (Table 4). In line with this, ALAT circulating levels were also significantly higher in the saline group than in rats receiving GHRP-6 (Table 4). In addition to focal necrosis, perivascular fibrotic induration (Figure 5G) was another hepatic change attributable to Dox cumulative toxicity and evidenced in the saline group. Interestingly, no evidences of pathologic fibrotic induration were ever detected in the liver of rats receiving GHRP-6 (Figure 5H). Small intestine, specially jejunum and ileum walls were also impacted by Dox administration. Intestinal damages included scattered foci of transmural necrosis in saline group which were consistently prevented by GHRP-6 administration (not shown). Kidneys were affected by Dox toxicity. Figure 5I illustrates tubular epithelial cells with cytoplasmic ballooning and nuclear pyknosis, which contrasts with the protective effect exerted by GHRP-6 treatment, in which most of the nuclei exhibit normal aspect (Figure 5J). The quantification of the tubular system damages in the saline group indicated the significant prevalence of lethal irreversible changes, as compared to the effect of GHRP-6. Similarly, the coagulative necrosis found in most microscopic fields of the bronchial epithelium in the saline group was broadly reduced by the GHRP-6 intervention (Table 4).

**TABLE 4:** Quantification of Dox-induced damages in extra-cardiac organs.

Variable	Saline	GHRP-6	*p*
Passive hepatic congestion (degree)	2.75 ± 0.27	0.69 ± 0.59	< 0.0001
Serum ALAT (U/L)	81.38 ± 12.73	29.38 ± 5.8	< 0.0001
Damaged renal tubules (%)	78.41 ± 12.43	20.53 ± 7.20	< 0.0001
Damaged bronchi (%)	70.95 ± 14.82	42.98 ± 11.50	0.0028

Data are expressed as mean ± SD., Statistical analyses were performed using unpaired Student’s *t*-test.

### GHRP-6 intervention ameliorated oxidative stress cytotoxicity

**FIGURE 6:**
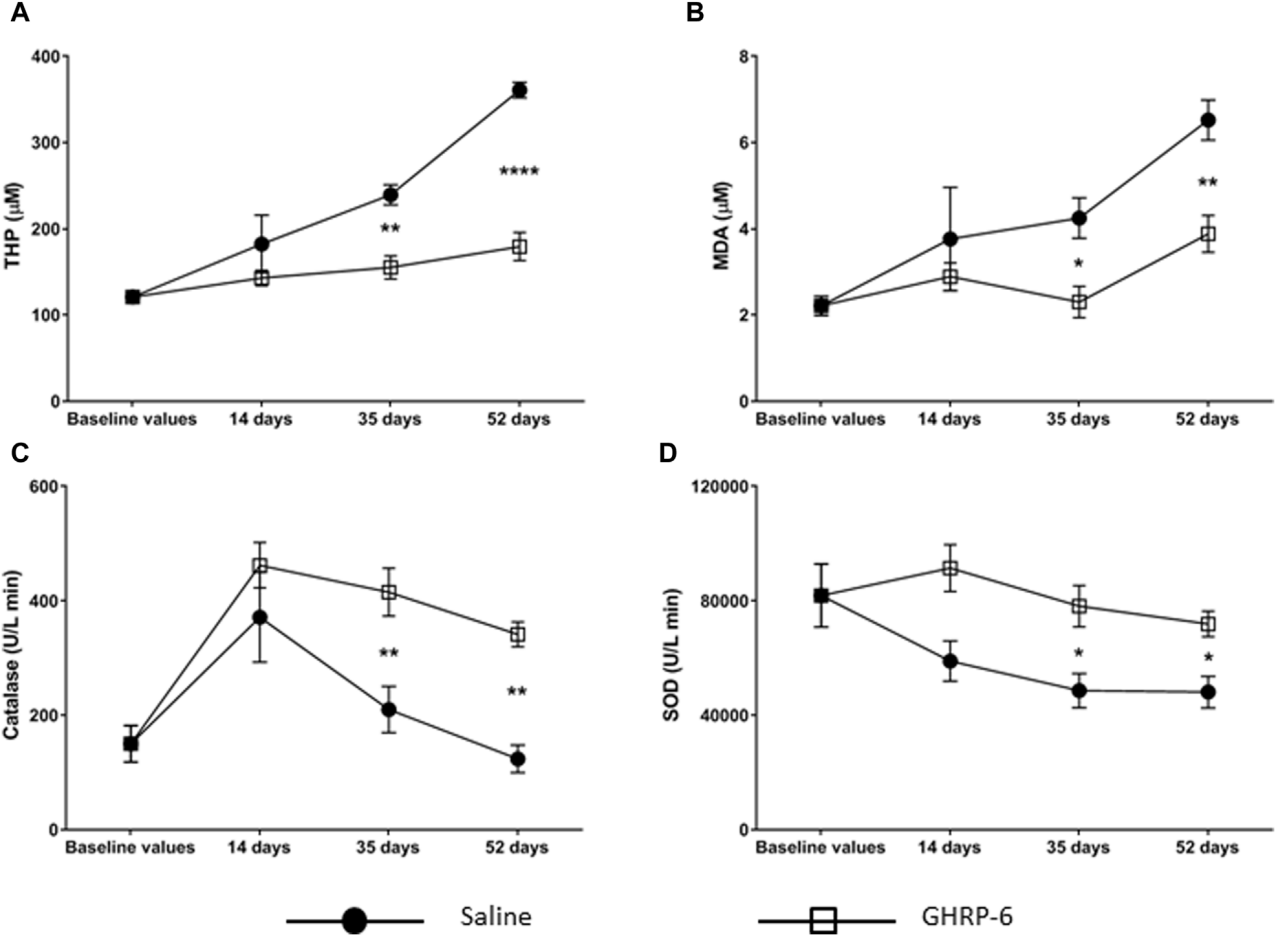
Serum oxidative stress markers in doxorubicin-treated animals. **(A)** Total hydroperoxides (THP). **(B)** Malondialdehyde (MDA). **(C)** Catalase. **(D)** Total superoxide dismutase (SOD). The values are represented as the mean ± SEM per experimental group. The statistical analyses were made by two-way ANOVA. Comparisons between treatment groups and among experimental time points were performed using Sidak’s and Dunnett’s multiple comparisons tests, respectively. *p < 0.05; **p < 0.01; ****p < 0.0001 represents statistical differences between the Saline and GHRP-6 groups at the different time points.

The profile of the serum redox markers studied is shown in Figure 6. Dox administration provoked a progressive increase in the THP and MDA concentrations of the saline group (*p* < 0.05 in both cases) (Figures 6A, B). This increase was significantly higher than that produced in the GHRP-6 counterpart for both parameters on day 35 (THP: *p* = 0.0010; MDA: *p* = 0.0442) and day 52 (*p* < 0.0001 and *p* = 0.0039, respectively). Catalase levels showed a biphasic behavior (Figure 6C). During the first 14 days of Dox administration, the activity of this enzyme progressively increased in both saline (*p* = 0.0052) and GHRP-6 (*p* < 0.0001) groups with respect to baseline values. However, with increasing Dox cumulative levels, catalase activity became inhibited, which was far more pronounced in the saline rats than in those treated with GHRP-6 in both day 35 (*p* = 0.0022) and day 52 (*p* = 0.0088). Serum SOD activity became gradually depressed in correspondence with Dox administration time in saline group (*p* < 0.05 for days 35 and 52). Nevertheless, concomitant GHRP-6 intervention positively impacted in SOD enzyme activity preservation, as compared to baseline values (*p* > 0.05) and to saline group (Figure 6D, day 35: *p* = 0.0462; day 52: *p* = 0.0363).

### GHRP-6 stimulated the expression of a survival gene

**FIGURE 7:**
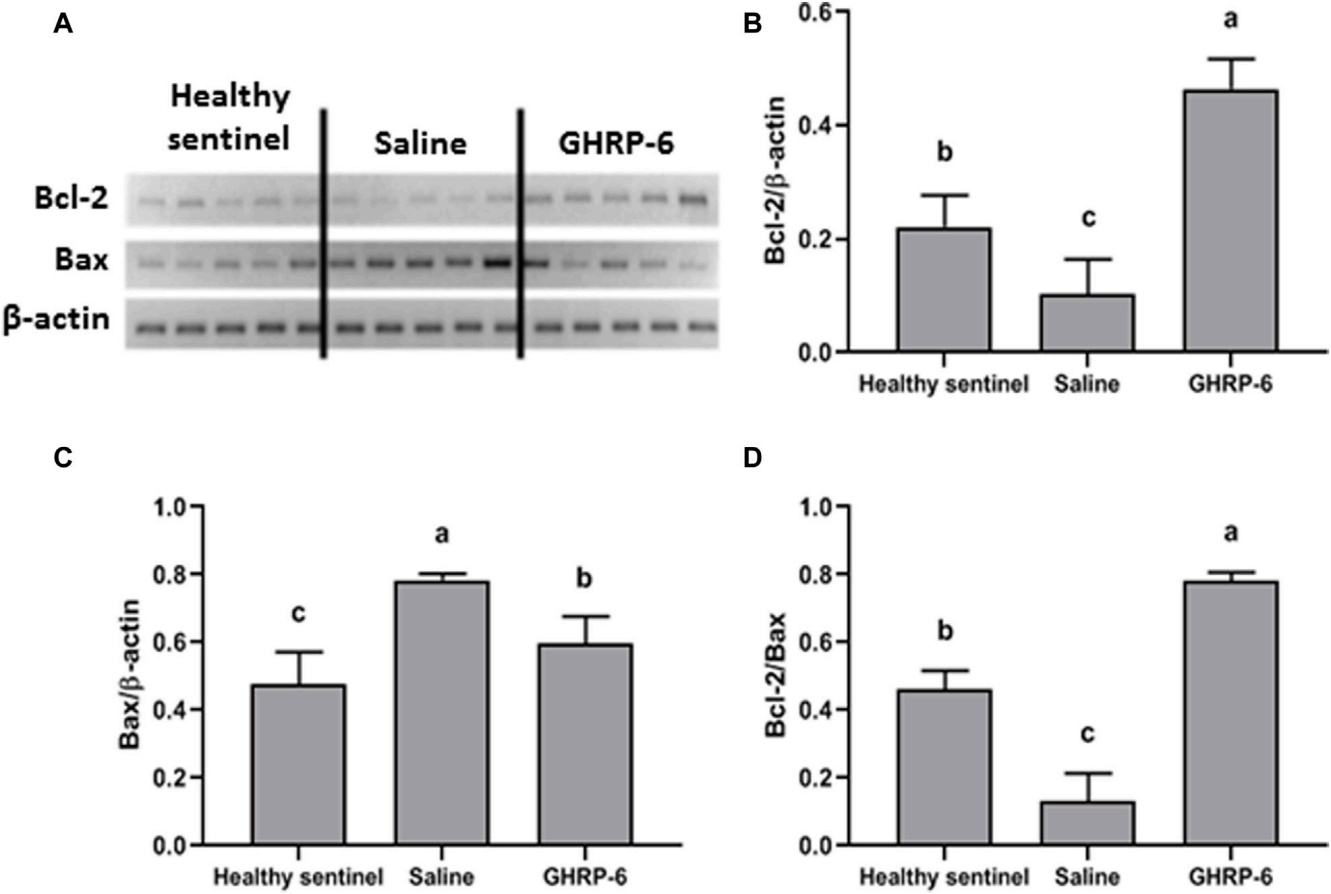
Influence of GHRP-6 in apoptosis regulatory gene in healthy and doxorubicin-treated groups. **(A)** Bands obtained by RT-PCR technique. Results normalized to β-actin gene expression are shown in the graphs: **(B)** Bcl-2; **(C)** Bax; **(D)** The Bcl-2/Bax ratio representing the balance between pro-survival and pro-apoptosis gene expression levels in favor to Bcl-2 expression. Bars indicate the mean ± SD (*n* = 5) per group. For multiple statistical comparisons, a one-way ANOVA followed by the Newman Keuls multiple comparison test were used. Significant statistical differences among the three experimental groups (*p* < 0.05) are denoted by different letters **(a,b,c)**.

GHRP-6 concomitant intervention produced a significant increase in Bcl-2 myocardial expression with a simultaneous reduction of the pro-apoptotic Bax (for both *p* < 0.001), as compared to healthy sentinel rats, and definitely in relation to saline group. In the latter, Bax exhibited the largest expression level (*p* < 0.001). The maximal value of Bcl2/Bax ratio calculated for the GHRP-6 group significantly exceeded those estimated for the sentinel and the saline groups (*p* < 0.05) (Figure 7).

### GHRP-6 treatment contributed to preserve cardiomyocytes organelles

**FIGURE 8:**
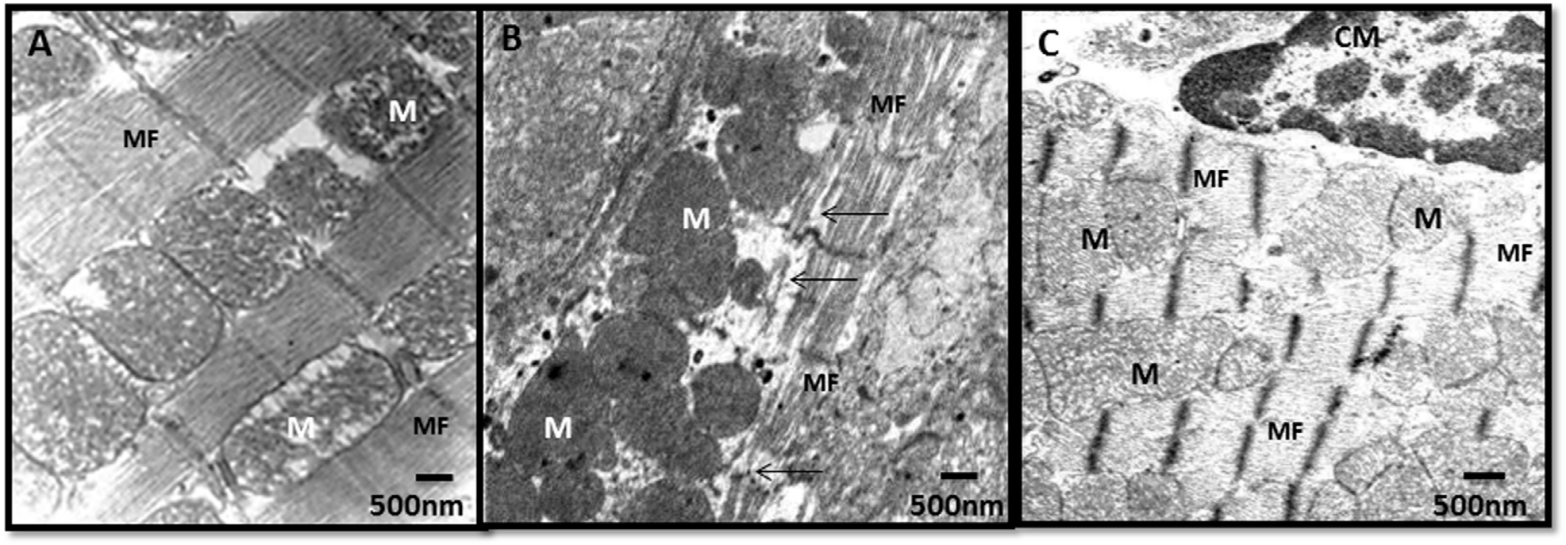
Myocardial ultrastructural characterization in samples of healthy and doxorubicin-treated rats. **(A)** Healthy sentinel control. The normal ultrastructure of the cardiac muscle is shown with myofibrils (MF) and mitochondria (M) within normal limits. **(B)** Saline. Loss of continuity of the myofibrils (arrows) and electron-dense mitochondria with loss of their cristae. **(C)** GHRP-6. Cardiomyocytes (CM), myofibrils, and mitochondria with cristae within normal limits. Bar: 500 nm.

Dox administration caused cardiomyocytes sarcolemmal vacuolization, myofibrils fragmentation, and mitochondrial damages that included membranes dilation, matrix ballooning, and cristae fragmentation and disappearance. Figure 8A shows the ultra-structural aspect of the myocardium of sentinel healthy animals. In contrast with this, Figure 8B is representative of the damages observed in the saline group, consistent on cardiomyocytes fragmentation and mitochondrial matrix damages. GHRP-6 intervention accounted for a noticeable sarcolemmal and mitochondrial structural preservation (Figure 8C).

## Discussion

Dox has been known since late 1960s, and its use in the oncological armamentarium remains as a first-line drug for the treatment of a wide variety of cancers of epithelial and non-epithelial origin. Nonetheless, its short and long-term cardiotoxic effects are of foremost clinical significance (Podyacheva and Toropova, 2022). Myriad of molecules have been historically investigated with the expectation to neutralize specific or multiple targets within the pleomorphic cardiomyocyte damaging cascade (Dulf et al., 2023). After all these efforts, dexrazoxane is the only currently approved treatment to prevent Dox-induced cardiotoxicity. In the meantime, cardioprotective prophylaxis strategies stand as an urgent requirement for cancer patients to prevent antineoplastic therapy disruption, and consequently reduce mortality (Wang et al., 2021).

The accumulated Dox dose of 30 mg/kg as an inducer of severe, progressive, and chronic cardiotoxicity was adopted from previous experiences (To et al., 2003). Since our pilot experiments, this dose proved to trigger an outspoken morbid state of DCM/CHF, characterized by echocardiographic signs of ventricular dilation, walls thinning, and LV mechanical failure. The heart failure involved an increase in both systolic and diastolic volumes, and the well-known distal complications as right side passive congestion with peritoneal, hepatic, and pulmonary expression. The fact that our echocardiographic, biochemical, and morphological findings are in line with previous descriptions of the experimental system (Shivakumar et al., 2012; Subburaman et al., 2014; Sandamali et al., 2019; Khedre et al., 2021), support and validate the practical usefulness of our model. Consequently, we examined on this scenario the hypothesis that GHRP-6 in concomitant administration to Dox, may prophylactically attenuate the myocardial and multiorgans changes associated to DCM/HF, and to the anthracycline intrinsic toxicity. The GHRP-6 dose used in this study had already evidenced cardioprotective effects by reducing infarct size over 70% in a porcine model of acute myocardial infarction (Berlanga et al., 2007). As described, GHRP-6 administration in parallel to Dox prevented cardiomyocytes fibrils consumption and ventricular cavities dilation, which accounted for an effective preservation of the LV systolic activity. This GHRP-6 mediated preservation of the systolic function, likely contributed to prevent extracardiac expressions of HF, as hepatic venous congestion and alveolar septal flooding. Comprehensively speaking, GHRP-6 concomitant administration to Dox attenuated cardiac and extracardiac toxicity, reducing animals’ morbidity and mortality. This intervention however did not show to avert animals’ cachexia. Preceding findings indicated that GHSR1a agonistic stimulation increases appetite and improves body weight gain (Yuan and Wang, 2020).

A previous study had already shown that GHRP-6 (100 μg/kg/day) administered during 4 weeks, prevented DCM in a completely different, non-toxic substrate, based in TO hamsters with an inborn deficiency in the myocardial δ-sarcoglycan (Iwase et al., 2004). In consonance with it, GHRP-2 (1 mg/kg), another synthetic GH secretagogue analog to GHRP-6, and ligand for GHS-R1a and CD36 (Titterington et al., 2009) also attenuated progressive LV remodeling and systolic dysfunction in the same TO hamster line (Kato et al., 2010).

Two relevant effects were displayed by GHRP-6 intervention in the realm of Dox-derived toxicity: (Schultheiss et al., 2019): the broad cytoprotective responses induced by the peptide in different internal epithelial organs, and (Riehle and Bauersachs, 2019) the anti-fibrotic response detected in both liver and kidney parenchyma. GHRP-6 intervention showed to rescue from coagulative necrosis a variety of epithelial cells as hepatocytes, kidneys tubular cells, bronchial epithelia, and the jejunum-ileum enterocytes. In relation to GHRP-6 extracardiac cytoprotective profile, the only precedent study we are aware of, revealed that a single prophylactic injection with GHRP-6 aborted the onset of Curling-like ulcers, and luminal bleeding in stressed conscious rats. Interestingly, GHRP-6 displayed a dual protective mechanism, one by directly activating survival signalers on the gastric epithelial cells, while simultaneously blunting the vagal efferent function, reducing the stress-stimulated gastric acid production (Guo et al., 2012). The anti-fibrotic effect, a previously described observation by our group appears to be driven by a transcriptional downregulation of TGF-β1 and CTGF, and by counteracting the accumulation and formation of extracellular matrix ingredients and fibroblasts cytoskeleton organization proteins (Berlanga-Acosta et al., 2012; Fernandez-Mayola et al., 2018).

Altogether these data support the notion that these therapeutic bounties are driven by the agonistic stimulation of myocardial and extra-myocardial CD36 and/or GHSR1a receptors by activating survival pathways, downregulating fibrogenic cytokines, and optimizing energetic homeostasis shunts that cooperate in cellular survival (Hosoda, 2022; Shu et al., 2022; Glatz et al., 2023). Whether these cytoprotective and anti-fibrotic effects are solely consequent to the stimulation of GHS-R1a has been debated (Yuan et al., 2021) and is beyond the scope of our study. However, this is a pharmacologically-relevant issue that deserves comment. A line of experimental evidences based on cardiac and non-cardiac cells in which GHS-R1a activation has been aborted through chemical antagonism and genetic silencing, incites to conclude that this receptor is irreplaceable for the activation of salvage kinases, as for the anti-fibrotic, anti-oxidant, anti-inflammatory, and proangiogenic effects upon its occupation by acylated ghrelin (Shati and El-Kott, 2019; Wang et al., 2020; Zhang and Xie, 2020; Shati and El-Kott, 2021; Wang et al., 2023). However, the early observation by Bladanzi and co-workers in which both ghrelin and des-acyl ghrelin inhibited apoptosis in H9c2 cardiomyocytes which do not express GHSR1ainaugurated the alternative line, sustaining that prosurvival pathways may be triggered and functionally activated through a GHSR-independent pathway (Baldanzi et al., 2002). More recent studies have demonstrated that des-acyl ghrelin exhibits cardioprotective activities and anti-fibrotic effects through a GHSR independent pathway (Pei et al., 2014; Liu et al., 2020). Accordingly, Delhanty and co-workers have indicated that unacylated ghrelin is a physiological component of the circulation (Delhanty et al., 2015), behaves like a separate hormone (Delhanty et al., 2012), and that it is likely endowed with its own receptor (Delhanty et al., 2013). Likewise, the potential contribution of CD36 on the light of our findings demands consideration. First, GHRP-6 binds CD36 while the recently generated azapeptide analogues retain high and selective binding affinity for CD36 (Proulx et al., 2020), second, CD36 plays a key role in providing the myocardium with its major energy substrate (Shu et al., 2022), third, agonistic binding of CD36 protected against myocardial damage and dysfunction by ischemia/reperfusion (Bessi et al., 2012), fourth, mice deficient for CD36 exhibit reduced tolerance to myocardial ischemia/reperfusion injury (Irie et al., 2003), whereas humans harboring inborn CD36 mutations, exhibit a variety of heart diseases including hypertrophic cardiomyopathy, dilated cardiomyopathy, or coronary heart disease (Tanaka et al., 2001), and fifth, alike GHSR-1a, CD36 is represented in a broad constellation of mammals tissues and organs (Zibara et al., 2002; Jacome-Sosa et al., 2021), including those that appeared protected by GHRP-6 in our experiment. Hence, further studies are warranted in order to selectively discern the role of each candidate receptor.

A thorough blueprint of the Dox-associated toxic mechanism is yet to be depicted (Pugazhendhi et al., 2018). However, it seems that mitochondrial damage/dysfunction with the ensued uncontrolled generation of reactive oxygen species (ROS), is as proximal trigger in the cascade of Dox-induced cardiomyocytes harms (Montalvo et al., 2020; Nordgren and Wallace, 2020; Kong et al., 2022). As described by others (Dulf et al., 2023; Shi et al., 2023), we observed that Dox administration was characterized by progressive increase of hydroperoxides and reactive aldehydes formation, along with a depletion of major neutralizing anti-oxidant enzymes. Concomitant GHRP-6 administration significantly counterbalanced these events, while keeping catalase and SOD enzymes significantly active. This is not an unexpected finding since we had observed that GHRP-6 reduced myocardial injury in a porcine model of AMI, by the preservation of antioxidant defense systems and the ensuing decrease in ROS pool (Berlanga et al., 2007). Others also demonstrated that ghrelin, the unique endogenous ligand of GHSR-1a, prevented neuronal apoptosis in a model of hypoxia/ischemia by attenuating oxidative stress and enhancing other survival drivers as Sirt1, PGC-1α, UCP2 (Huang et al., 2019). Mitochondria are identified as the major source of ROS and a main determinant of Dox-induced cardiac damages. Disruption of mitochondrial physiology contributes to alter metabolic and redox circuits in cardiac cells, ultimately culminating in increased apoptosis (Wallace et al., 2020). We therefore deem that the GHRP-6 effect on cardiac mitochondria structural preservation is noteworthy, and may have definitively contributed to reduce oxidative reactants spillover.

Since previous studies (Kagan et al., 2009) invoked Dox accumulation in cardiac mitochondria as primary trigger of apoptosis, we evaluated the expression ratio of Bcl-2/Bax genes. Interestingly, cardiac Bcl-2 expression associated to GHRP-6 administration exceeded the physiological expression level detected in the healthy sentinel group. Early pharmacological characterizations of GHSR1a ligands showed to inhibit cardiomyocyte apoptosis in a rat model of chronic heart failure (Xu et al., 2005). This GHRP-6 mediated antiapoptotic ability was further confirmed and extended through other *in vitro* and *in vivo* models (Paneda et al., 2003; Delgado-Rubin et al., 2009; Granado et al., 2011), which appeared mediated via the PI3 K/Akt/Bcl-2 salvage pathway (Yuan and Wang, 2020). Conclusively, Dox-associated myocardial cells demise may have been at least partially prevented by Bcl-2 upregulation, along with a concomitant decrease of Bax expression.

Although this work is limited for not having acquired cardiovascular/pulmonary hemodynamic constants, gasometry readings, or examined the integrity of the intestinal wall barrier function, for a more comprehensive evaluation of GHRP-6 cytoprotective spectrum; it provides the first evidences on the cardiac and extracardiac protective effect of a peptidyl GH secretagogue against an anti-neoplastic anthracycline. It also offers a foundational platform for subsequent cardio-and-cytoprotection studies within the cardio-oncology realm.

Although with an underlying controversial substrate; ghrelin, GHSR1a, and CD36 appear implicated in the multistep process of malignant transformation, and cancer cells progression and metastasis (Soleyman-Jahi et al., 2019; Guerrero-Rodriguez et al., 2022; Kotta et al., 2022). This may introduce regulatory constrains for the subsequent investigational development of these candidates. Nonetheless, oncology remains orphan of pharmacological tools that may act as “life-saving drugs” for empty niches as cancer-associated cachexia-anorexia syndrome, and chemotherapy-associated cardiotoxicity. Both processes may cause precocious mortality (Yeom and Yu, 2022; Dempke et al., 2023). Accordingly, we deem that GHRP-6 and/or other GHS “drugability” is justified, which will subsequently entail the clinical use of these candidates under the medical personalized analysis of risk-benefit balance.

## Significance

Dilated cardiomyopathy (DCM) is a poor-prognosis condition characterized by ventricular dilation with evolving deterioration of the systolic function ultimately leading to heart failure (HF). Cancer patients treated with the chemotherapeutic anthracycline Doxorubicin (Dox) are frequently affected by the drug-related myocardial toxicity. This condition leads to chemotherapy scheme discontinuation and therefore to cancer progression. Despite continuous research efforts, DCM mortality rates are high, remaining as one of the leading causes of heart transplantation. This study confirms and extends that the classic growth hormone (GH) secretagogue GHRP-6, is endowed with potent cardio and cyto-protective abilities. In an experimental model of Dox-induced DCM, GHRP-6 concomitant administration prevented ventricular myofibrils consumption, dilation, and accordingly preserved within physiological limits left ventricle ejection fraction. Globally speaking, GHRP-6 reduced rats’ morbidity and mortality in a significant manner as compared to saline control. Furthermore, GHRP-6 also exerted a broad cytoprotective effect by reducing the thresholds of parenchymal necrosis and apoptosis in most epithelial internal organs. The treatment proved to abort in this system the fibrotic induration in liver, kidneys, and lungs as a consequence of Dox-toxicity. From a mechanistic perspective, GHRP-6 attenuated the pro-oxidant arm and enhanced the anti-oxidant reserves before Dox-challenge, attenuated mitochondrial matrix-ultrastructural damages, and increased the expression of Bcl-2 as antiapoptotic gene. This is the first evidence on the cardiac and extracardiac protective effects of a peptidyl GH secretagogue against an anti-neoplastic anthracycline. GHRP-6 candidacy as cardioprotective drug is further supported by its broad safety and tolerability profile upon its parenteral administration.

## Figures and Tables

**Table:** 

**DCM**	dilated cardiomyopathy
**LV**	left ventricle
**LVEF**	left ventricle ejection fraction
**HF**	heart failure
**Dox**	doxorubicin
**DNA**	deoxyribonucleic acid
**GH**	growth hormone
**GHRP-6**	GH releasing peptide 6
**GHSR1a**	GH secretagogue receptor 1a
**CD36**	cluster differentiation 36
**IGF-1**	insulin like growth factor type 1
**CENPALAB**	National Center for Laboratory Animals Breeding
**CIGB**	Center for Genetic Engineering and Biotechnology
**LVDd**	LV diastolic diameter
**LVSd**	LV systolic diameter
**IVSs**	inter-ventricular septum thickness in systole
**LVWs**	LV posterior wall thickness in systole
**TEM**	transmission electron microscopy
**THP**	total hydroperoxides
**MDA**	malondialdehyde
**SOD**	superoxide dismutase
**ALAT**	alanine amino transferase
**Bcl-2**	Abbreviation of B cell Lymphoma. The gene product family plays promotes cell survival
**Bax**	Also identified as bcl-2-like protein 4 with pro-apoptotic effect
**RNA**	Ribonucleic acid
**RT-PCR**	Reverse transcription-polymerase chain reaction
**ANOVA**	analysis of variance
**CHF**	congestive heart failure
**TGF-β1**	Transforming growth factor beta 1
**CTGF**	Connective tissue growth factor
**Fas**	Pro-apoptotic product
**ROS**	reactive oxygen species
**AMI**	Acute myocardial infarction
**Sirt1**	silent mating type information regulation 2 homolog. Regulator of metabolism, cell death, aging, oxidative stress defense, and inflammation
**PGC-1α**	peroxisome proliferator-activated receptor γ coactivator-1 α. Broad regulator of mitochondrial life cycle and function, metabolism and cell survival
**UCP2**	Uncoupling protein 2, involved in oxidative stress prevention
**PI3 K**	phosphatidylinositol 3-kinases. A family of proteins involved in cell growth, proliferation, differentiation, motility, and survival
**Akt**	Protein kinase B. It is involved in cell cycle progression, survival, protein synthesis, and cell growth

## Data Availability

The original contributions presented in the study are included in the article/Supplementary material, further inquiries can be directed to the corresponding author.
